# Vanillin modulates activities linked to dysmetabolism in psoas muscle of diabetic rats

**DOI:** 10.1038/s41598-021-98158-7

**Published:** 2021-09-21

**Authors:** Veronica F. Salau, Ochuko L. Erukainure, Kolawole A. Olofinsan, Omamuyovwi M. Ijomone, Nontokozo Z. Msomi, Md.Shahidul Islam

**Affiliations:** 1 Department of Biochemistry, University of KwaZulu-Natal, Westville Campus, Durban, 4000 South Africa; 2 Department of Pharmacology, University of the Free State, Bloemfontein, 9300 South Africa; 3 Department of Biochemistry, Veritas University, Bwari, Abuja, Nigeria; 4 Department of Human Anatomy, School of Health and Health Technology, Federal University of Technology, Akure, Nigeria

**Keywords:** Endocrine system and metabolic diseases, Biochemistry, Metabolomics

## Abstract

Skeletal muscles are important in glucose metabolism and are affected in type 2 diabetes (T2D) and its complications. This study investigated the effect of vanillin on redox imbalance, cholinergic and purinergic dysfunction, and glucose-lipid dysmetabolism in muscles of rats with T2D. Male albino rats (Sprague–Dawley strain) were fed 10% fructose ad libitum for 2 weeks before intraperitoneally injecting them with 40 mg/kg streptozotocin to induce T2D. Low (150 mg/kg bodyweight (BW)) and high (300 mg/kg BW) doses of vanillin were orally administered to diabetic rats. Untreated diabetic rats and normal rats made up the diabetic control (DC) and normal control (NC) groups, respectively. The standard antidiabetic drug was metformin. The rats were humanely put to sleep after 5 weeks of treatment and their psoas muscles were harvested. There was suppression in the levels of glutathione, activities of SOD, catalase, ENTPDase, 5′Nucleotidase and glycogen levels on T2D induction. This was accompanied by concomitantly elevated levels of malondialdehyde, serum creatine kinase-MB, nitric oxide, acetylcholinesterase, ATPase, amylase, lipase, glucose-6-phosphatase (G6Pase), fructose-1,6-biphophastase (FBPase) and glycogen phosphorylase activities. T2D induction further resulted in the inactivation of fatty acid biosynthesis, glycerolipid metabolism, fatty acid elongation in mitochondria and fatty acid metabolism pathways. There were close to normal and significant reversals in these activities and levels, with concomitant reactivation of the deactivated pathways following treatment with vanillin, which compared favorably with the standard drug (metformin). Vanillin also significantly increased muscle glucose uptake ex vivo. The results suggest the therapeutic effect of vanillin against muscle dysmetabolism in T2D as portrayed by its ability to mitigate redox imbalance, inflammation, cholinergic and purinergic dysfunctions, while modulating glucose-lipid metabolic switch and maintaining muscle histology.

## Introduction

Type 2 diabetes (T2D) ranks among the most common types of diabetes as it has been linked to over 90% of mortality and morbidity associated with diabetes^1^. It is typified by poor utilization of insulin secreted by the pancreatic β-cells, causing perturbation in the metabolisms of carbohydrates, proteins, and fatty acids and in turn leads to hyperglycemia^2^. This has been associated with persistent β-cell dysfunction and insulin resistance, leading to glucotoxicity and lipotoxicity in cells^3^.

Aside the pancreas, skeletal muscles also play pivotal functions in glucose metabolism, as they participate in the uptake of dietary glucose in healthy individuals with normal glucose tolerance^4^. In fed states, the pancreas secrets insulin that facilitates glucose uptake in skeletal muscles which is used for muscle energy production. Thereby reducing postprandial increase in blood glucose level. While in the fasting states, skeletal muscle glucose uptake is reduced and the muscle switches to free fatty acids (FFAs) for energy production^4,5^. These normal activities are however impaired in T2D owing to diminished pancreatic insulin secretion and/or insulin resistance, thus culminating in decreased muscle glucose uptake, impaired muscle glucose-lipid metabolic homeostasis, suppressed muscle glycogen levels, perturbed muscle mitochondrial oxidative phosphorylation and muscle cholinergic dysfunction^5–7^.

Excessive utilization of FFAs and their dysmetabolism over glucose utilization by skeletal muscles in T2D has been involved in the pathogenesis and progression of muscle insulin resistance^8,9^. Increased hyperglycemia-induced production of free radicals and/or perturbed oxidative phosphorylation in skeletal muscle mitochondria overwhelms the muscles’ intrinsic antioxidant system. Thus, leading to oxidative stress which has also been linked to insulin resistance^10,11^. Purinergic activities have been reported for their importance in normal physiological activities of skeletal muscles due to their expression of purinergic receptors, P2X5, P2Y_1_ and P2X2^12^. These receptors are activated by nucleoside produced from phospho-hydrolysis of the purines such as Adenosine triphosphate (ATP) and Adenosine monophosphate (AMP) by purinergic enzymes. Altered activities of purinergic enzymes have been implicated in muscle dysfunction leading to impaired contraction and glucose uptake^13,14^. These factors contribute greatly to skeletal muscle fiber atrophy, sarcopenia and myopathy in the long run^15,16^.

A major therapeutic mechanism of some antidiabetic drugs like the biguanides such as metformin involves improving muscle glucose uptake and improvement of muscle functions^17,18^, while modulating glucose-lipid metabolic switch^5^. However, there are concerns about the side effects and tolerance associated with the use of biguanides which include abdominal spasm, lactic acidosis, metallic aftertaste, nausea and diarrhea^19,20^. This has encouraged an increased search for alternative treatments with little or no side effects.

**Figure 1:**
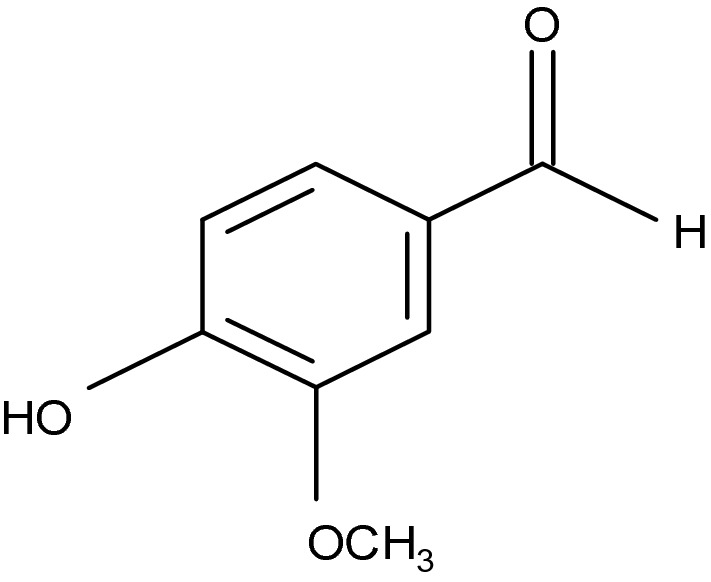
Chemical structure of vanillin.

The diabetic properties of phenolics and their ability to improve muscle glucose uptake has been documented^5,21^. Their antioxidant potency have been linked to these activities and other medicinal properties of phenolics^21,22^. Vanillin (4-hydroxy-3-methoxybenzaldehyde; Fig. 1) ranks among the common phenolics which is widely utilized as flavor in food, cosmetics and pharmaceutical industries^23^. It is a benzaldehyde with of methoxy and hydroxy functional groups at positions 3 and 4 respectively. Vanillin has been documented for its antidiabetic^24^, anticancer^25^, anti-sickling^26^, antimutagenic^27^, and neuroprotective activities^23^.

Despite its reported antidiabetic activities, there is still a dearth of literature on the therapeutic mechanism of vanillin against T2D and its complications. Thus, this study was aimed at investigating the ability of vanillin to arrest oxidative insult, lipid dysmetabolism, cholinergic and purinergic dysfunctions, while improving glucose metabolism and mitigating myopathy in psoas muscles of type 2 diabetic rats.

## Materials and methods

### Vanillin

Vanillin (≥ 99.8% HPLC pure) was supplied by Sigma-Aldrich chemical company, Johannesburg, South Africa.

### Animal ethical clearance

Animal ethical clearance for this research study was authorized by the Animal Research Ethics Committee (AREC) of the University of KwaZulu-Natal, Durban, South Africa (Ethical protocol approval number: AREC/022/019D). All methods were performed in accordance with the relevant guidelines and regulations of the University of KwaZulu-Natal AREC, Durban, South Africa. All animal studies followed the arrive guidelines.

### Animals

Thirty (30) Sprague–Dawley male albino rats (220 ± 30 g) were obtained from the Biomedical Research Unit (BRU), University of KwaZulu-Natal, Durban, South Africa. Animals were supplied standard rat chow and water ad libitum and allowed to acclimatize for 1 week under standard 12 h light/dark cycle.

### Experimental groups and type 2 diabetes induction

Animals were divided into six experimental groups comprising 5 rats each, namely: **NC**: Normal control (untreated non-diabetic rats); **DC**: Diabetic control (untreated diabetic rats); **DVL**: Diabetic rats administered low dose (150 mg/kg body weight [BW]) of vanillin; **DVH**: Diabetic rats administered high dose (300 mg/kg BW) of vanillin; **DMT**: Diabetic rats administered 200 mg/kg BW metformin; and **NVX**: Non-diabetic rats administered high dose (300 mg/kg bw) of vanillin.

After 7 days acclimatization, T2D was induced in the rats, by employing an earlier established protocol^28^. The diabetic groups (DC, DVL, DVH and DMT) were administered 10% fructose ad libitum for two (2) weeks, while ordinary water was supplied to the NC and NVX (normal) groups. Following an overnight fast, rats in the diabetic groups were intraperitoneally (i.p) injected with 40 mg/kg BW streptozotocin dissolved in 0.1 M citrate buffer (pH 4.5), whereas animals in the normal groups were only injected with citrate buffer. Exactly 7 days after streptozotocin injection, the non-fasting blood glucose (NFBG) concentrations of all the animals were measured with a glucometer (Glucoplus, Glucoplus Inc., Quebec, Canada). Rats in diabetic groups having blood glucose concentration ˃200 mg/dL were considered as diabetic.

### Treatment

A gavage needle was used to orally administer the respective doses of treatment to each animal once daily for 5 weeks. Animals in DVL group were administered low (150 mg/kg BW) vanillin dose, while those in DVH were administered with high (300 mg/kg BW) dose. The animals in NVX group were equally received the high dose (300 mg/kg BW) of vanillin, whereas the animals in DMT group were administered with 200 mg/kg BW of metformin. The control groups (NC and DC) were only administered with distilled water. The doses of vanillin were selected based on the previous studies on its toxicity in rats^29^.

### Sacrifice and collection of blood and muscles

At the end of the treatment, animal were humanely put to sleep by euthanizing with isofor after an overnight fast. Blood was collected into plain centrifuge tubes through cardiac puncture. Psoas muscles from each animal were harvested and rinsed in 0.9% NaCl to get rid of blood stains. For histology, about 0.3 g of the harvested muscle was fixed in 10% buffered formalin. A 0.5 g of each harvested muscle was homogenized in 5 mL phosphate buffer solution with 1% triton X-100 (50 mM; pH 7.5). The homogenates were then centrifuged at 20,000 g for 10 min (4°C). The resulting supernatants were decanted into 2 mL Eppendorf tubes, labelled accordingly, and preserved at − 80°C for subsequent analysis.

### Determination of oxidative stress markers

Oxidative stress markers of the muscle tissues were analyzed in the supernatant by determining reduced glutathione (GSH) level^30^, catalase^31^ and superoxide dismutase (SOD)^32^ activities, and malondialdehyde (MDA) level^33^.

#### Reduced glutathione (GSH) level

A 300 μL of muscle tissue supernatant was deproteinized in 300 μL 10% TCA and then centrifuged at 3500 rpm for 5 min. Then 200 μL of the supernatant was added to 50 μL of Ellman’s reagent in a 96 well plate and incubated for 10 min on ice. Absorbance was read at 415 nm. GSH level was extrapolated from a plotted GSH standard curve.

#### Superoxide dismutase (SOD) activity

A 15 μL of muscle tissue supernatants was added to 170 μL of 0.1 mM diethylenetriaminepentaacetic acid (DETAPAC) in a 96-well plate. At the point of reading, a freshly prepared 15 μL of 1.6 mM 6-hydroxydopamine was added, the plate was gently swirled, and the absorbance was immediately recorded at 492 nm for 3 min at 1 min interval.

#### Catalase activity

This was determined by incubating 10 μL of the muscle tissue supernatant with 340 μL of sodium phosphate buffer (50 mM; pH 7.0) at room temperature for 5 min. Then 150 μL of 2 M hydrogen peroxide was added and absorbance was measured at 240 nm at 1 min interval for 3 min.

#### Malondialdehyde (MDA) level

Lipid peroxidation was estimated by boiling a mixture containing 200 μL of tissue supernatant, 200 μL of 8.1% SDS solution, 750 μL of 20% acetic acid, 2 mL of 0.25% thiobarbituric acid and 850 μL of miliQ water for 1 h. The mixture was allowed to cool and 200 μL was pipetted in a 96-well plate. The absorbance was read at 532 nm. The malondialdehyde (MDA) level of tissues was extrapolated from an MDA standard curve.

### Determination of nitric oxide (NO) level

The NO levels of muscle tissues were determined using previously described method of Yoon et al.^34^ with some modifications as described by Erukainure et al^35^. Briefly, an equal volume (100 μL) of muscle tissue supernatant or distilled water (blank) and Griess reagent was incubated in a dark chamber at 25°C for 30 min. Absorbance was read at 548 nm. The NO level of the tissues was extrapolated from a standard curve of sodium nitrite (NaNO_2_).

### Determination of cholinergic activity

The cholinergic activity of the muscle tissues was determined by assaying for acetylcholinesterase activities in the tissue supernatant using the Ellman’s method^36^. A 40 μL of muscle tissue supernatant was incubated with 20 μL of Ellman’s reagent (3.3 mM, pH 7.0) and 100 μL of 0.1 M phosphate buffer (pH 8) for 20 min at room temperature. Then 20 μL of 0.05 M acetylcholine (substrate) was added. The absorbance was measured at 412 nm at 1 min intervals for 3 min.

### Determination of purinergic activities

To determine purinergic activities of the muscle tissues, adenosine triphosphatase (ATPase), ecto-nucleoside triphosphate diphosphohydrolase (ENTPDase), and 5′nucleotidase (5′NT) activities were analyzed in the tissue supernatant.

#### ATPase activity

ATPase activity was determined based on a previously described method^37^ with slight modifications as described by Erukainure et al.^38^ Briefly, 200 μL of muscle tissue supernatant was incubated with 200 μL 5 mM KCl, 1300 μL of 0.1 M Tris–HCl buffer, and 40 μL of 50 mM ATP in a shaker for 30 min at 37°C. Then 1 mL of distilled H_2_O, followed by 1.25% ammonium molybdate were added to terminate the reaction. A freshly prepared 1 mL of ascorbic acid (9%) was then added to the reaction mixture and incubated for 30 min at room temperature. Absorbance was read at 660 nm and ATPase activity was expressed as the amount of inorganic phosphate (Pi) liberated/min/mg of protein.

#### ENTPDase activity

The ENTPDase activity was determined using a previously described method^39^. A mixture containing 20 μL of muscle tissue supernatant and 200 μL of the reaction buffer (1.5 mM CaCl_2_, 5 mM KCl, 0.1 mM EDTA, 10 mM glucose, 225 mM sucrose and 45 mM Tris–HCl) was incubated for 10 min at 37°C. Then 20 μL of 50 mM ATP was then added and incubated for 20 min at 37°C in a shaker. The reaction was terminated with 10% TCA. After 10 min incubation on ice, the absorbance was measured at 600 nm and the enzyme activity was expressed as the amount of inorganic phosphate (Pi) liberated/min/mg of protein.

#### 5’nucleotidase activity

5′NT activity was analyzed based on a previously established method^40^. Briefly, 20 μL supernatant was incubated with 50 μL 0.1 M MgCl_2_ and 50 μL of 0.1 M Tris–HCl for 10 min at 37°C. Then 20 μL of 50 mM ATP was added and further incubated for 20 min at 37°C. Reaction was stopped with 200 μL of 10% TCA, incubated on ice for 10 min and absorbance was measured at 600 nm. The enzyme activity was calculated as the amount of inorganic phosphate (Pi) liberated/min/mg of protein.

### Determination of carbohydrate metabolizing enzymes activities

The carbohydrate metabolizing enzymes activities of muscle tissues were determined by analyzing the supernatants for glucose-6-phosphatase (G6Pase), fructose-1,6-bisphosphatase (FBPase), glycogen phosphorylase and amylase activities as described below.

#### Glucose 6 phosphatase activity

This was determined based on a previously described method^38^ with slight modifications^41^. A 200 μL of muscle tissue supernatant was incubated with 50 mM ATP, 0.25 M glucose, 5 mM KCl, and 0.1 M Tris–HCl buffer in a shaker for 30 min at 37°C. The reaction was stopped by adding 1 mL of distilled water and 1.25% ammonium molybdate. A freshly prepared 9% ascorbic was then added and incubated for another 30 min at room temperature. The absorbance was measured at 660 nm and G6Pase activity was expressed as the amount of inorganic phosphate (Pi) liberated/min/mg of protein.

#### Fructose 1,6 bisphosphatase activity

Fructose-1,6-bisphosphatase activity was determined according to the method described by Balogun and Ashafa^42^ with slight modification. A 100 μL of the supernatant was incubated with 1200 μL of 0.1 M Tris–HCl buffer (pH 7.0), 100 μL of 0.1 M KCl, 250 μL 0.1 M MgCl_2_, 100 μL of 0.05 M fructose, and 250 μL of 1 mM EDTA for 15 min at 37°C. The reaction was stopped by adding 10% TCA and centrifuged for 10 min at 3000 rpm (4°C). Then 100 μL of the supernatant was added to 50 μL of 1.25% ammonium molybdate and a freshly prepared 9% ascorbic acid in a 96-well plate and incubated for 20 min at room temperature. Absorbance was read at 680 nm and the activity of the enzyme was expressed as the amount of inorganic phosphate (Pi) liberated/min/mg of protein.

#### Glycogen phosphorylase activity

This was determined according to the method described by Balogun and Ashafa^42^. Briefly, a mixture of 100 µL of the muscle tissue supernatant, 64 mM glucose-1-phosphate and 4% glycogen was incubated for 10 min at 30°C. The reaction was stopped with 20% ammonium molybdate in concentrated H_2_SO_4_. It was further incubated for 45 min at 30°C following an addition of Elon reducer and distilled water. Absorbance was measured at 340 nm using a Synergy HTX Multi-mode reader and the enzyme activity was expressed as amount of inorganic phosphate (Pi) liberated/min/mg of protein.

### Amylase activity

This was carried out by employing a previously established protocol^43^, with slight modifications^44^. Briefly, 50 µL of muscle tissue supernatant was incubated with equal volume of starch (1%) dissolved in phosphate buffer for 30 min at 37°C. Then 200 µL of dinitrosalicylate (DNS) reagent was added to the mixture and boiled for 10 min. After cooling, the absorbance was measured at 540 nm at 1 min interval. Amylase activity was expressed as the rate of reaction (ΔA/min).

### Determination of muscle glycogen content

The muscle glycogen content was estimated according to a previously established protocol^45^ with slight modification. A 0.5 g of muscle tissue was digested in 0.5 mL 30% KOH saturated in Na_2_SO_4_. The mixture was boiled for 30 min and completely immersed in ice to cool. Then 670 µL of 95% ethanol was added to the mixture and centrifuged for 30 min at 840 g. This procedure was repeated for proper precipitation of the glycogen content. The supernatant was discarded, and the precipitate was dissolved with 1 mL of distilled water. A 20 µL aliquot of the dissolved precipitate (glycogen) was made up to 200 µL with distilled H_2_O.Then 200 µL of 5% phenol was added to the 200 µL dissolved glycogen or standard followed by a rapid and careful addition of 1 mL of concentrated H_2_SO_4_. The mixture was vortexed, boiled for 10 min and cooled. The absorbance was measured at 490 nm and the glycogen content was extrapolated from a standard curve.

### Determination of lipase activity

This was determined in the muscle tissues using previously established method^46^ with slight modifications^5^. Briefly, 100 μL of the muscle tissue supernatant and 169 μL of Tris buffer (pH 7.0) was incubated for 15 min at 37°C. This was followed by an addition of 5 μL of 10 mM p-nitrophenyl butyrate in dimethyl formamide (p-NPB) and an incubation of 15 min at 37°C. The absorbance was measured at 405 nm at 1 min interval. Lipase activity was expressed as the rate of reaction (ΔA/min).

### Serum CK-MB analysis

The collected blood was made to stand in ice, 2 h before centrifuging at 2,000 g. The serum was collected and analysed for CK-MB level, with an automated Chemistry Analyzer (Labmax Plenno, Labtest Co. Ltd., Lagoa Santa, Brazil), using commercial assay kit according to the procedure in the manufacturer’s manual.

### Histopathology examination

Formalin fixed muscle tissues were processed and fixed on slides. The slides were deparaffinized and subjected to hematoxylin and eosin staining. Images were observed and captured using a digital brightfield microscope (OMAX 40-2000X 3MP Digital Compound Microscope, USA).

### Extraction of muscle lipid metabolites

The lipid metabolites of the muscle tissues were extracted for GC–MS metabolic profiling by employing previously established protocol, with slight modification^47^. Briefly, 0.5 g of muscle tissues was homogenized in 5 mL of cold chloroform and centrifuged at 20,000 g for 10 min at 4°C following an incubation on ice for 20 min. The resultant supernatants were collected into HPLC vials and subjected to GC–MS profiling to identify the metabolites.

### GC–MS profiling of extracted muscle lipid metabolites

The extracted muscle lipid metabolites were subjected to GC–MS profiling on an Agilent technologies 6890 Series GC coupled with (an Agilent) 5973 Mass Selective detector and driven by Agilent chemstation software. A HP-5MS capillary column was utilized to separate the metabolites. Injections of 1 μL of the samples were made in splitless mode. Other working parameters were: **Carrier gas**: ultra-pure helium; **Flow rate**: 60 mL h^−1^; **Initial oven temperature**: 60°C for 2 min; **Final oven temperature**: 285°C at the rate of 5°C min^-1^ with a hold time of 3 min; **Ion source temperature**: 230°C; **Quadrupole temperature**: 150°C; **Electron ionization mode and electron multiplier voltage:** 70 eV and 1859 V. The metabolites were identified with the aid of an inbuilt NIST mass spectral library.

### Data normalization

GC–MS identified lipid metabolites and their values were subjected to normalization by sample median, cube root transformation and mean centering using the MetaboAnalyst 5.0 online server (https://www.metaboanalyst.ca/). The box plots and kernel density plots before and after normalization are presented in Fig. S1.

### Pathway analysis

Pathway enrichment analysis was used to identify relevant pathways of the identified muscle lipid metabolites in the effect of vanillin on lipid metabolism in muscles of diabetic rats using the MetaboAnalyst 5.0 online server^48^.

### Ex vivo studies

#### Animals

Five (5) Sprague Dawley male rats with average weights of 200 ± 20 g were collecetd from the Biomedical Research Unit (BRU), University of KwaZulu-Natal, Durban, South Africa. The animals were fasted overnight and humanely sacrificed by euthanizing with isofor. The muscle was excised and immediately analyzed for ex vivo glucose uptake activity.

#### Glucose uptake studies in isolated rat psoas muscle

The glucose uptake stimulatory effect of vanillin was determined in isolated psoas muscles of rats, using a previously described method^49^ with slight modifications. A 0.5 g of the freshly harvested rat psoas muscle tissues were rinsed in Krebs buffer solution. Muscle tissues were placed in 8 mL of Krebs buffer with different concentrations of vanillin (30–240 μg/mL) and 11.1 mM of glucose. The reaction mixture was incubated for 2 h under a 5% CO_2_, 95% oxygen and 37°C conditions. Metformin served as the standard drug while the control was an incubation with ‘glucose only’ without vanillin or metformin.

The extent of glucose uptake was determined from the glucose concentration of the Krebs buffer before and after incubation using an Automated Chemistry Analyzer (Labmax Plenno, Labtest Inc., Lagoa Santa, Brazil) and was calculated as expressed below.

$${\mathrm{Glucose\, uptake\, per\, gram\, of \,rat\, psoas\, muscle}}=\frac{GC1-GC2}{Weight\, of\, muscle\, tissue\, (g)}$$

where GC1 and GC2 represent glucose concentrations in mg/dL before and after incubation respectively**.**

### Molecular docking study

The 3D x-ray diffraction structure of GLUT 4 protein with ID: 3PCU and resolution of 2.00 Å was retrieved from Protein Data Bank. Dock prep tool of UCFS Chimera software V. 1.14^50^ was used to remove water molecules co-crystallized with the protein. Then, hydrogen atoms were added, followed by protonation states and gasteiger charges^51^. Avogadro V1.2^52^ was used to obtain the optimal global configuration of vanillin downloaded from PubChem before ligand preparation as outlined earlier with the protein. Docking simulation was performed with AutodockVina V1.1.2^53^, which uses a Lamarckian Genetic Algorithm. The docking was done with a search volume of coordinate X = 10, Y = 14, Z = 8 and box spacing of 1 Å. BIOVIA Discovery Studio^54^ was employed to investigate the amino acid interactions at the active site of the ligand–protein complex with the lowest root mean square deviation (RMSD) value of 1.496.

### Statistical analysis

Results are presented as mean ± SD and were analyzed using one-way analysis of variance (ANOVA). The Tukey’s HSD-multiple range post-hoc test was used to derive significant differences between means at *p* < 0.05. The statistical analysis was performed with the IBM Statistical Package for the Social Sciences (SPSS) for Windows, version 23.0 (IBM Corp., Armonk, NY, USA). The GC–MS identified lipid metabolites were subjected to clustering analysis which covers for heat maps and principal component analysis (PCA) using the MetaboAnalyst 5.0 online server (https://www.metaboanalyst.ca/).

## Results

**Figure 2:**
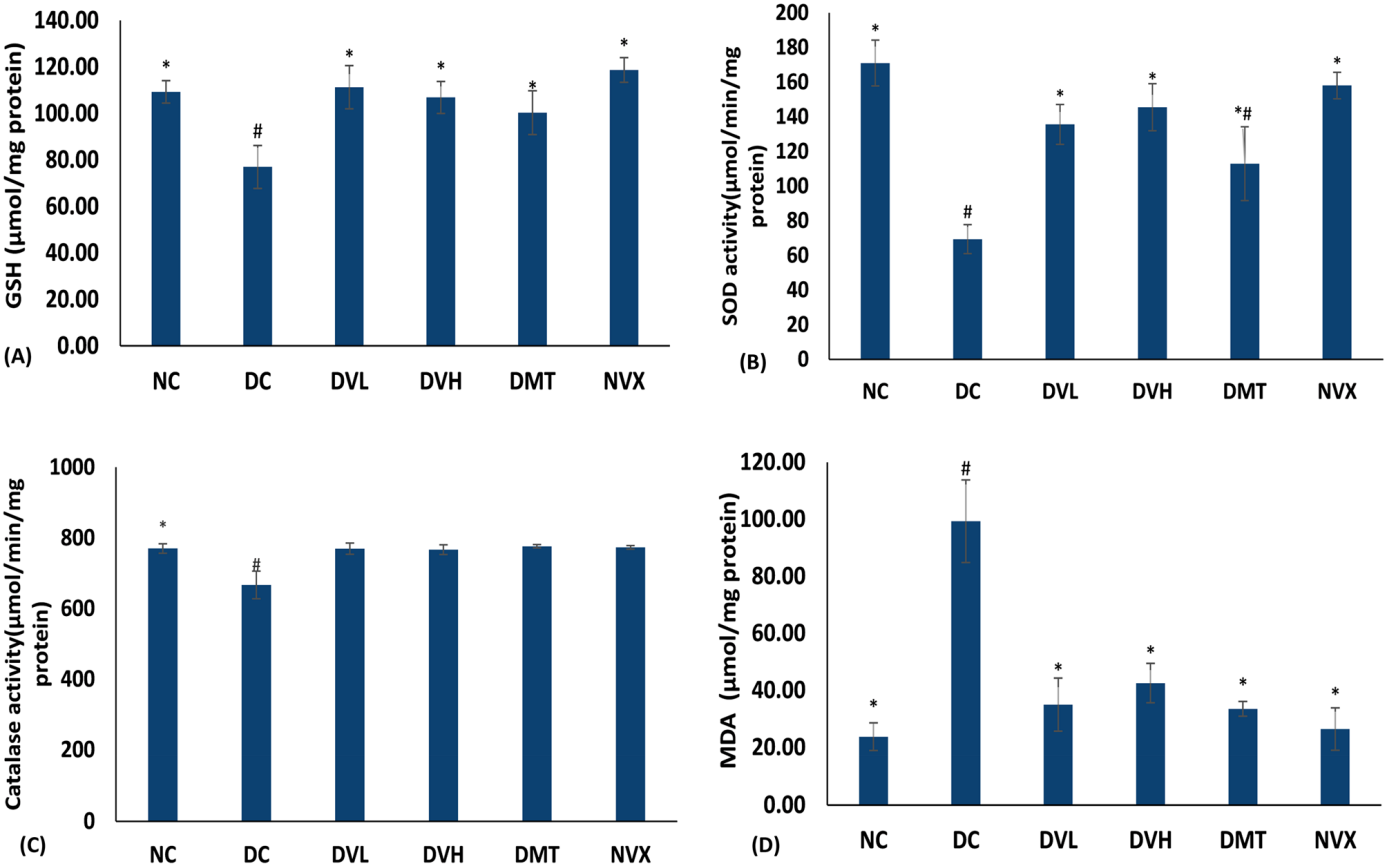
Antioxidant status of experimental groups. Value = mean ± SD; n = 5. *Statistically significant (*p* < 0.05) to DC; #Statistically significant (*p* < 0.05) to NC. NC = normal rats, DC = diabetic control, DVL = diabetic rats + vanillin (150 mg/kg b.w), DVH = diabetic rats + vanillin (300 mg/kg bw), DMT = diabetic rats + metformin (200 mg/kg b.w), and NVX = normal rats + vanillin (300 mg/kg bw).

As represented in Fig. 2A–D, T2D induction resulted in significant (*p* < 0.05) depletion in GSH level, SOD and catalase activities in muscle tissues, with concomitant elevation of MDA levels. Vanillin treatment at both low and high doses, significantly reversed these activities and levels as depicted by the elevated GSH level, SOD and catalase activity, while suppressing MDA level.

**Figure 3:**
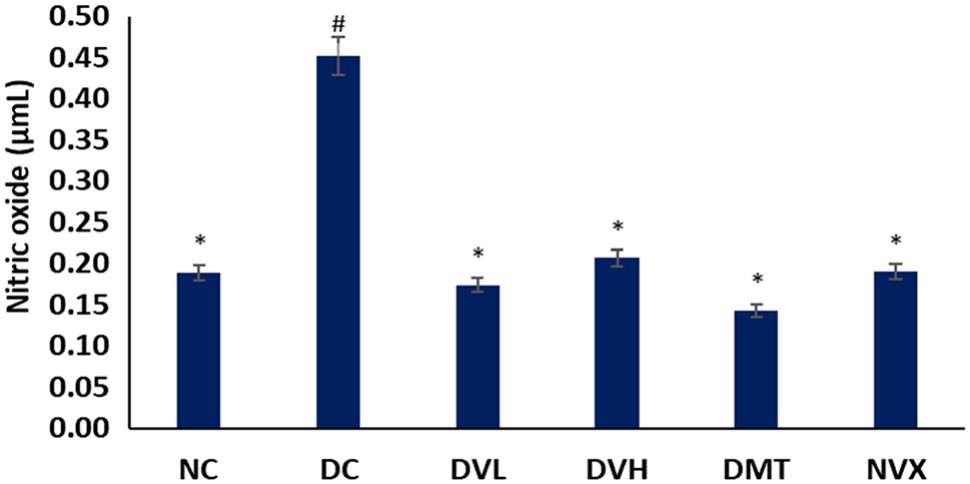
Nitric oxide levels of experimental groups. Value = mean ± SD; n = 5. *Statistically significant (*p* < 0.05) to DC; #Statistically significant (*p* < 0.05) to NC. NC = normal rats, DC = diabetic control, DVL = diabetic rats + vanillin (150 mg/kg b.w), DVH = diabetic rats + vanillin (300 mg/kg bw), DMT = diabetic rats + metformin (200 mg/kg b.w), and NVX = normal rats + vanillin (300 mg/kg bw).

T2D induction significantly (*p* < 0.05) increased the level of NO in muscle tissues as shown in Fig. 3. The level was depleted significantly (*p* < 0.05), after treatment with vanillin at both doses and compared favorably with the DMT (metformin-treated) and NC (normal control) groups.

**Figure 4:**
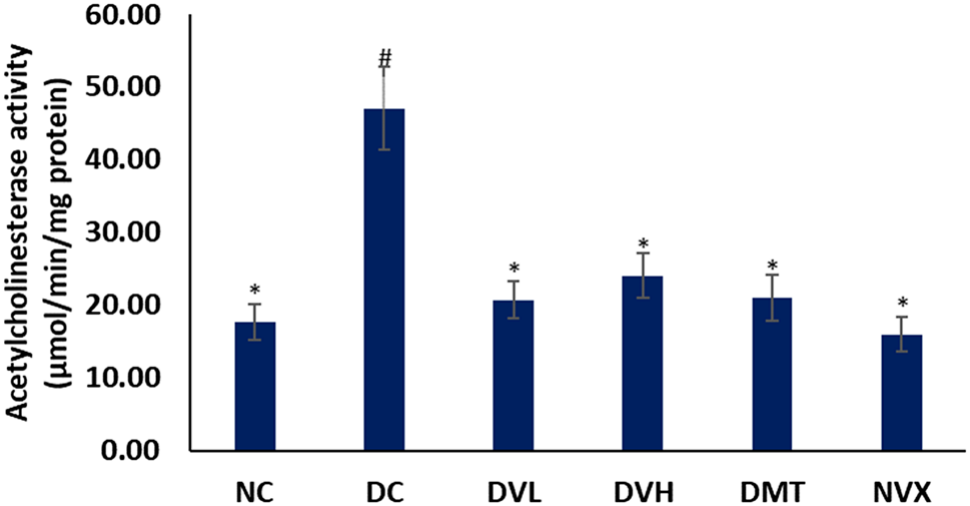
Acetylcholinesterase activities of experimental groups. Value = mean ± SD; n = 5. *Statistically significant (*p* < 0.05) to DC; #Statistically significant (*p* < 0.05) to NC. NC = normal rats, DC = diabetic control, DVL = diabetic rats + vanillin (150 mg/kg b.w), DVH = diabetic rats + vanillin (300 mg/kg bw), DMT = diabetic rats + metformin (200 mg/kg b.w), and NVX = normal rats + vanillin (300 mg/kg bw).

As presented in Fig. 4, the muscle’s acetylcholinesterase activity was significantly (*p* < 0.05) elevated following the induction of T2D. Treatment with vanillin significantly reduced the activity, with the lower dose having a slight better activity compared to the higher dose. Vanillin treatment at both doses, however compared favorably with metformin and the normal rats.

**Figure 5:**
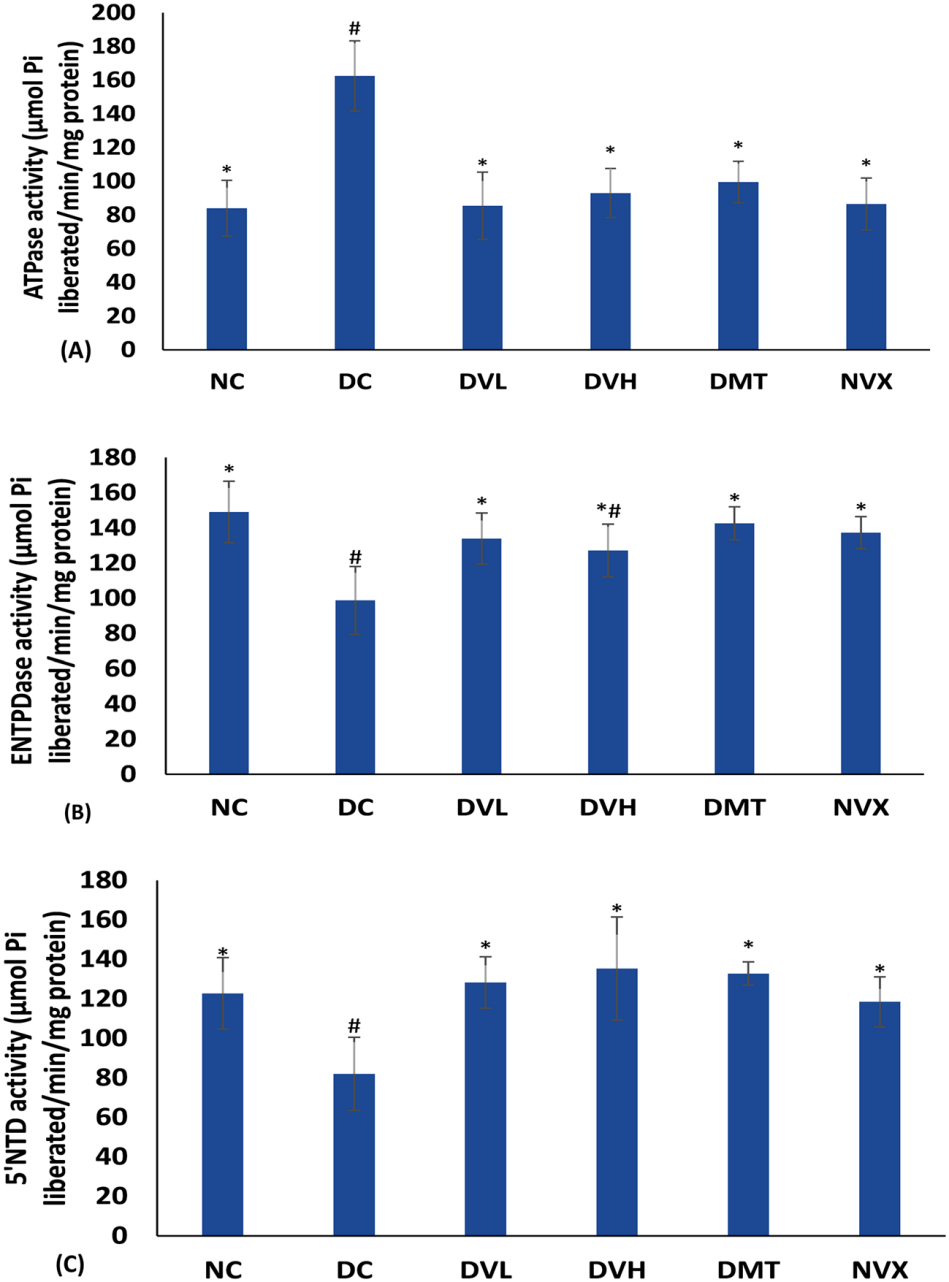
(**A**) ATPase, (**B**) E-NTPDase and (**C**) 5′NTD activities of experimental groups. Value = mean ± SD; n = 5. *Statistically significant (*p* < 0.05) to DC; #Statistically significant (*p* < 0.05) to NC. NC = normal rats, DC = diabetic control, DVL = diabetic rats + vanillin (150 mg/kg b.w), DVH = diabetic rats + vanillin (300 mg/kg bw), DMT = diabetic rats + metformin (200 mg/kg b.w), and NVX = Normal rats + vanillin (300 mg/kg bw).

Induction of T2D significantly (*p* < 0.05) elevated activity of ATPase in muscle tissues, while concomitantly reducing ENTPDase and 5′NT activities as shown in Fig. 5A–C. Treatment with vanillin significantly (*p* < 0.05) reversed these activities as portrayed by the reduced ATPase activity, and concomitant elevated activities of ENTPDase and 5′NT. Both doses vanillin compared favorably with metformin and the normal rats.

**Figure 6:**
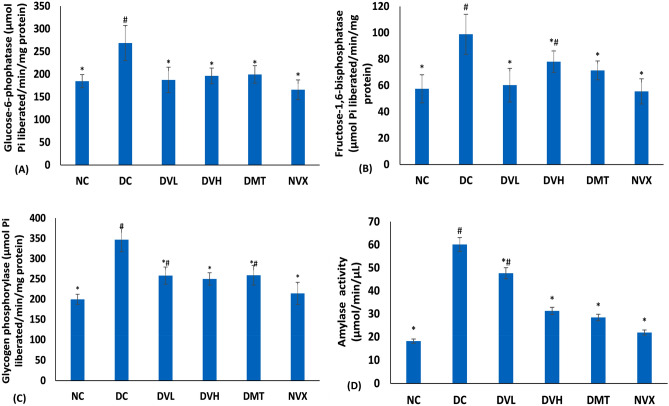
(**A**) Glucose 6-phosphatase, (**B**) FRUCTOSE-1,6-biphosphatase, (**C**) glycogen phosphorylase and (**D**) α-amylase activities of experimental groups. Value = mean ± SD; n = 5. *Statistically significant (*p* < 0.05) to DC; #Statistically significant (*p* < 0.05) to NC. NC = normal rats, DC = diabetic control, DVL = diabetic rats + vanillin (150 mg/kg b.w), DVH = diabetic rats + vanillin (300 mg/kg bw), DMT = diabetic rats + metformin (200 mg/kg b.w), and NVX = normal rats + vanillin (300 mg/kg bw).

The muscle G6Pase, FBPase, glycogen phosphorylase and amylase activities were significantly elevated on induction of T2D as shown in Fig. 6A–D. These activities were inhibited significantly (*p* < 0.05) to near normal after treatment with vanillin, with the low dose depicting better activities for glucose-6-phosphatase and fructose-1,6-biphosphatase while the high dose was more active on amylase inhibition.

**Figure 7:**
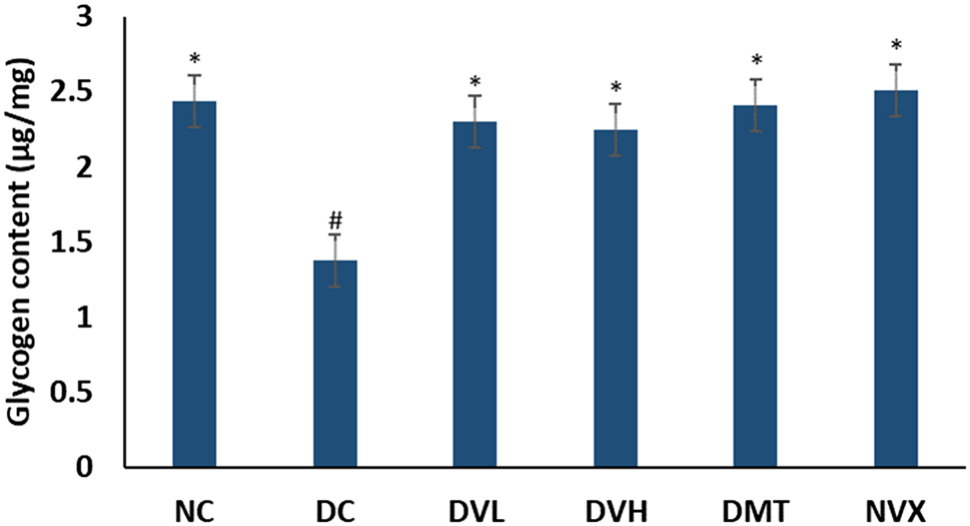
Glycogen content of experimental groups. Value = mean ± SD; n = 5. *Statistically significant (*p* < 0.05) to DC; #Statistically significant (*p* < 0.05) to NC. NC = normal rats, DC = diabetic control, DVL = diabetic rats + vanillin (150 mg/kg b.w), DVH = diabetic rats + vanillin (300 mg/kg bw), DMT = diabetic rats + metformin (200 mg/kg b.w), and NVX = normal rats + vanillin (300 mg/kg bw).

Induction of T2D led to significant (*p* < 0.05) depletion of muscle glycogen content as shown in Fig. 7. Treatment with vanillin at both doses significantly (*p* < 0.05) elevated the glycogen content and compared favorably with the DMT and NC groups.

**Figure 8:**
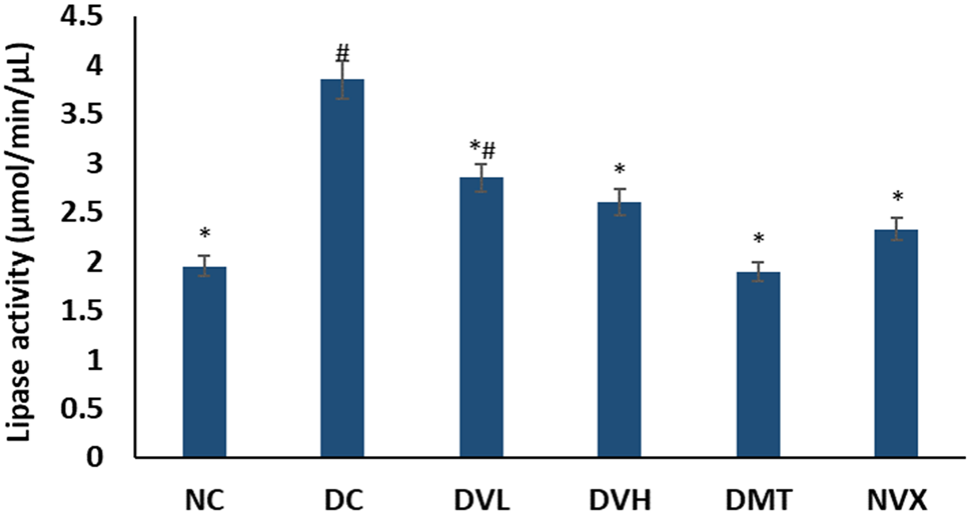
Lipase activities of experimental groups. Value = mean ± SD; n = 5. *Statistically significant (*p* < 0.05) to DC; #Statistically significant (*p* < 0.05) to NC. NC = normal rats, DC = diabetic control, DVL = diabetic rats + vanillin (150 mg/kg b.w), DVH = diabetic rats + vanillin (300 mg/kg bw), DMT = diabetic rats + metformin (200 mg/kg b.w), and NVX = normal rats + vanillin (300 mg/kg bw).

There was an elevation in the muscle lipase activity on induction of T2D as shown in Fig. 8. The activity was significantly (*p* < 0.05) suppressed in muscles of rats treated with vanillin, with the higher dose having a slight better activity. Vanillin treatment compared favorably with DMT and NC groups at both doses.

As represented in Table 1, induction of T2D led to 66.66% depletion of fatty acids, while concomitantly generating pentadecanoic acid, 9-octadecynoic acid and eicosanoic acid. Except for Eicosanoic acid, 2-hydroxyethyl ester, induction of T2D led to the complete depletion of fatty esters and fatty alcohols. It further led to the generation of steroids (2-Methylenecholestan-3-ol, Cholesta-4,6-dien-3-ol, Cholesteryl myristate and Cholesterol), and glycerol (Glycerol 1-palmitate). Treatment with vanillin at low dose led to the restoration of the depleted fatty acids except cis-13,16-Docasadienoic acid, with concomitant depletion of T2D-generated metabolites except pentadecanoic acid and eicosanoic acid. The high dose of vanillin restored only tetradecanoic acid and palmitoleic acid, while concomitantly generating (6Z)-6-Octadecenoic acid. Both doses of vanillin did not restore the degenerated fatty esters, but the low dose led to the generation of Hexanoic acid, tridecyl ester. n-Tetracosanol was the only fatty alcohol restored in the vanillin-treated diabetic rats. 2-Methylenecholestan-3-ol, Cholesta-4,6-dien-3-ol and Cholesteryl myristate were the steroid metabolites depleted on treatment with low dose of vanillin, while the high dose led to the depletion of 2-Methylenecholestan-3-ol and Cholesta-4,6-dien-3-ol. Interestingly, administration of vanillin to normal rats (NVX) led to major alterations in the lipid metabolites while generating 22-Dehydrocholesterol and 26,27-Dinorcholesta-5,22-dien-3-ol, (3.beta.,22E)-.

**Table 1:** GC–MS identified lipid metabolites in muscle tissues of experimental groups.

Classes	Metabolites	NC	DC	DVL	DVH	DMT	NVX
Fatty acid	Tetradecanoic acid	0.49 ± 0.03	ND	0.24 ± 0.01	0.25 ± 0.03	ND	ND
	Palmitoleic acid	0.77 ± 0.06	5.95 ± 1.05	1.01 ± 0.10	0.52 ± 0.02	6.32 ± 0.92	ND
	Hexadecanoic acid	10.30 ± 0.44	ND	9.62 ± 0.46	ND	ND	ND
	cis-13,16-Docasadienoic acid	3.20 ± 0.12	ND	ND	ND	ND	ND
	cis-13-Eicosenoic acid	6.51 ± 0.98	ND	9.88 ± 0.52	ND	ND	ND
	Octadecanoic acid	2.87 ± 0.23	0.31 ± 0.03	2.49 ± 0.17	2.57 ± 0.29	2.22 ± 0.18	ND
	Pentadecanoic acid	ND	7.38 ± 0.30	ND	7.49 ± 0.16	7.31 ± 0.86	6.50 ± 0.16
	9-Octadecynoic acid	ND	6.27 ± 0.34	3.33 ± 0.37	2.20 ± 0.07	ND	ND
	Eicosanoic acid	ND	2.59 ± 0.21	ND	ND	ND	ND
	(6Z)-6-Octadecenoic acid	ND	ND	ND	6.74 ± 0.82	ND	ND
	Decanoic acid	ND	ND	ND	ND	0.28 ± 0.02	ND
	cis-9-Hexadecenoic acid	ND	ND	ND	ND	0.88 ± 0.05	ND
	cis-13,16-Docasadienoic acid	ND	ND	ND	ND	2.42 ± 0.39	ND
Fatty ester	Eicosanoic acid, 2-hydroxyethyl ester	0.40 ± 0.03	0.41 ± 0.02	0.32 ± 0.02	0.38 ± 0.03	0.53 ± 0.02	ND
	2-Palmitoylglycerol	0.76 ± 0.18	ND	ND	0.37 ± 0.15	ND	ND
	Glycerin 1-tetradecyl ether	0.48 ± 0.10	ND	ND	ND	ND	ND
	Hexanoic acid, tridecyl ester	ND	ND	0.63 ± 0.32	ND	ND	ND
Fatty amide	Hydroxyethylpalmitamide	ND	ND	ND	0.25 ± 0.02		ND
Fatty alcohol	n-Tetracosanol-1	5.16 ± 0.69	ND	6.56 ± 1.44	5.82 ± 0.75	6.12 ± 1.50	ND
	1-Heneicosanol	3.70 ± 0.73	ND	ND	ND	ND	ND
	Tetraprenol	2.27 ± 0.04	ND	ND	ND	ND	ND
	2-Ethyl-2-methyl-1-tridecanol	ND	0.32 ± 0.03	ND	ND	ND	ND
Glycerol	Glycerol 1-palmitate	ND	0.18 ± 0.05	0.32 ± 0.03	ND	ND	ND
Glycol	Glycol myristate	0.31 ± 0.02	0.40 ± 0.02	ND	0.43 ± 0.03	0.35 ± 0.07	ND
Steroids	2-Methylenecholestan-3-ol	ND	0.36 ± 0.04	ND	ND	ND	ND
	Cholesta-4,6-dien-3-ol	ND	0.25 ± 004	ND	ND	ND	ND
	Cholesteryl myristate	ND	0.43 ± 0.05	0.38 ± 0.05	ND	ND	ND
	Cholesterol	ND	19.01 ± 5.64	13.25 ± 4.23	14.04 ± 3.80	24.21 ± 7.45	71.56 ± 0.86
	Squalene	0.41 ± 0.04	3.59 ± 0.12	4.21 ± 0.06	3.06 ± 0.07	3.48 ± 0.15	ND
	Retinal	ND	ND	ND	ND	0.37 ± 0.05	ND
	22-Dehydrocholesterol	ND	ND	ND	ND	ND	5.15 ± 0.29
	26,27-Dinorcholesta-5,22-dien-3-ol, (3.beta.,22E)-	ND	ND	ND	ND	ND	1.16 ± 0.27
Non-lipid	d-Mannitol, 1,1′-O-1,16-hexadecanediylbis-	ND	ND	0.23 ± 0.04	ND	ND	ND

Values = mean ± SD; n = 5. NC = normal rats, DC = Diabetic control, DVL = diabetic rats + vanillin (150 mg/kg b.w), DVH = Diabetic rats + vanillin (300 mg/kg bw), DMT = Diabetic rats + metformin (200 mg/kg b.w), and NVX = Normal rats + vanillin (300 mg/kg bw).

**Figure 9:**
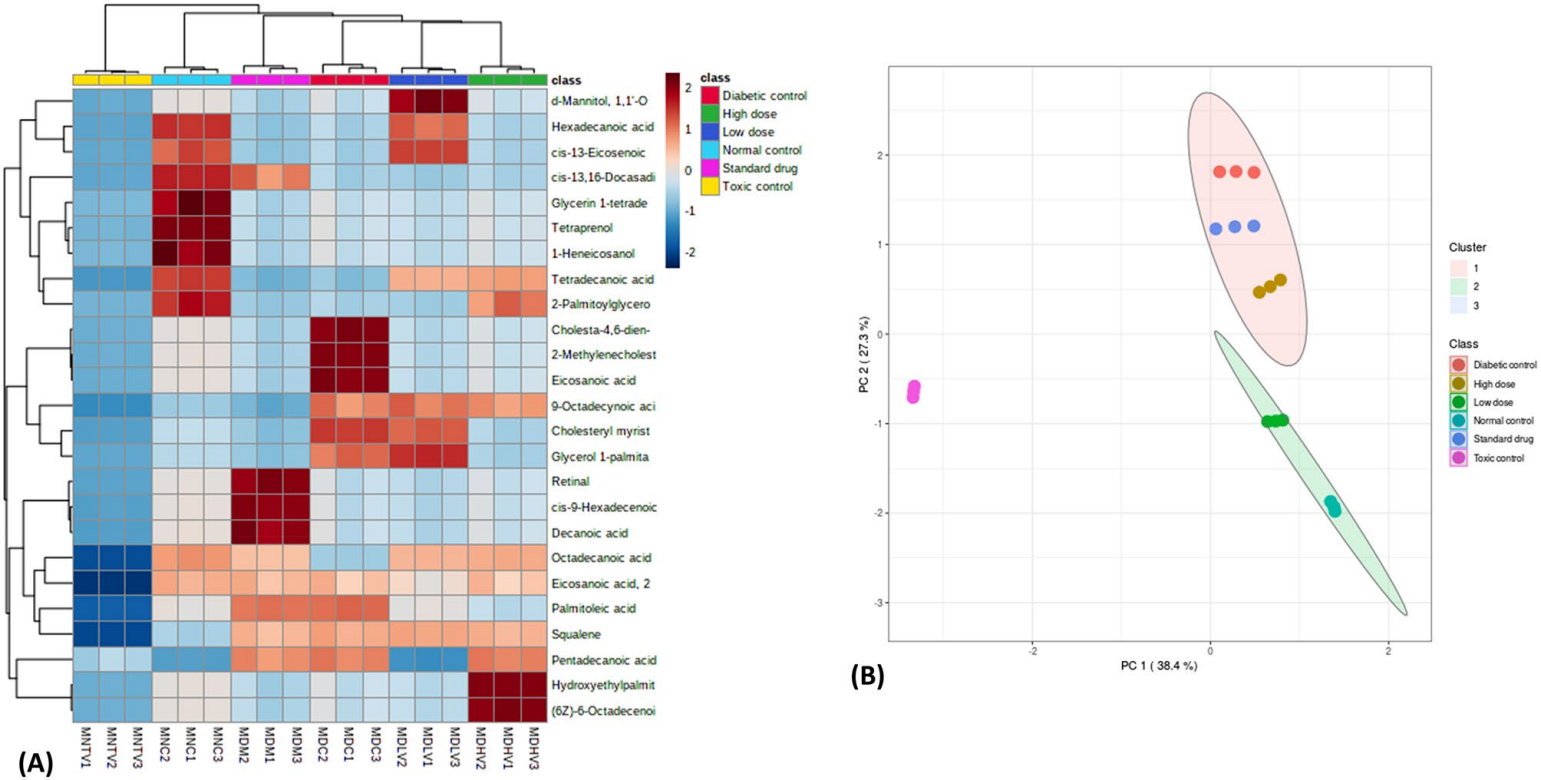
(**A**) Heat map; and (**B**) Score plot between the selected PCs of GC–MS identified lipid metabolites.

There were distinct changes in the GC–MS identified lipid metabolites and their spread across the experimental groups as depicted by the negative values and heat intensity as shown in Fig. 9A. Score plots between the selected PCs further corroborates these distinct changes and distributions as shown in Fig. 9B.

**Figure 10:**
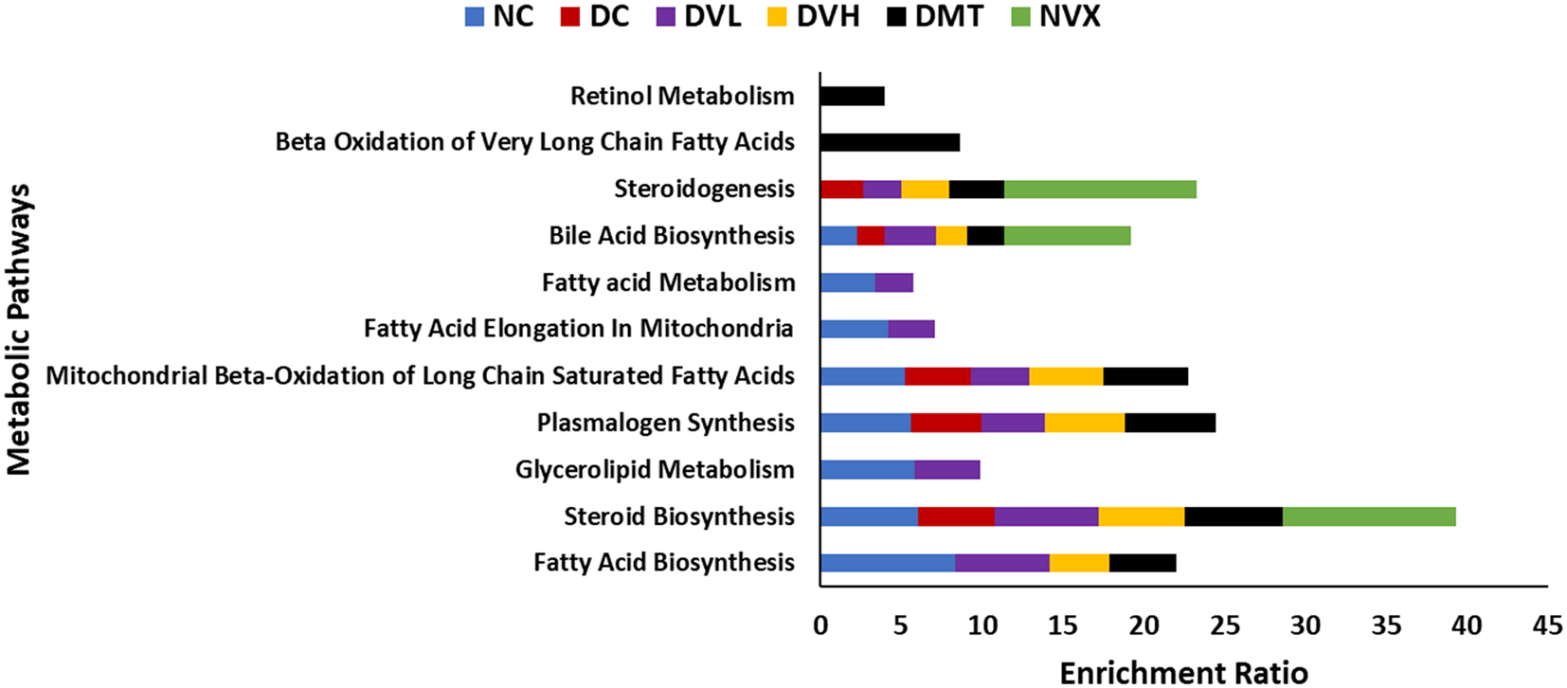
Enrichment ratio of identified pathways in experimantal muscle tissues. NC = normal rats, DC = diabetic control, DVL = diabetic rats + vanillin (150 mg/kg b.w), DVH = diabetic rats + vanillin (300 mg/kg bw), DMT = diabetic rats + metformin (200 mg/kg b.w), and NVX = normal rats + vanillin (300 mg/kg bw).

As shown in Table 2 and Fig. 10, pathway enrichment revealed that induction of T2D led to the inactivation of fatty acid biosynthesis, glycerolipid metabolism, fatty acid elongation in mitochondria, and fatty acid metabolism pathways, while activating steroidogenesis pathway. Treatment with vanillin at low dose led to reactivation of these pathways but had no effect on the T2D-activated pathway. High dose of vanillin only reactivated fatty acid biosynthesis pathway, with no effect on the T2D-activated pathway. Treatment with the standard antidiabetic drug, metformin led to the reactivation of fatty acid metabolism pathway, while concomitantly activating β oxidation of very long chain fatty acids and retinol metabolism pathways.

**Table 2:** Identified pathways in muscle tissues of experimental groups.

Pathway	NC	DC	DVL	DVH	DMT	NVX
Fatty acid biosynthesis	X	–	X	X	–	–
Steroid biosynthesis	X	X	X	X	X	X
Glycerolipid metabolism	X	–	X	–	–	–
Plasmalogen Synthesis	X	X	X	X	X	–
Mitochondrial β-oxidation of long chain saturated fatty acids	X	X	X	X	X	–
Fatty acid elongation in mitochondria	X	–	X	–	–	–
Fatty acid metabolism	X	–	X	–	X	–
Bile acid biosynthesis	X	X	X	X	X	X
Steroidogenesis	–	X	X	X	X	X
β oxidation of very long chain fatty acids	–	–	–	–	X	–
Retinol metabolism	–	–	–	–	X	–

X = present; – = not present. NC = NORMAL rats, DC = diabetic control, DVL = diabetic rats + vanillin (150 mg/kg b.w), DVH = Diabetic rats + vanillin (300 mg/kg bw), DMT = diabetic rats + metformin (200 mg/kg b.w), and NVX = Normal rats + vanillin (300 mg/kg bw).

**Figure 11:**
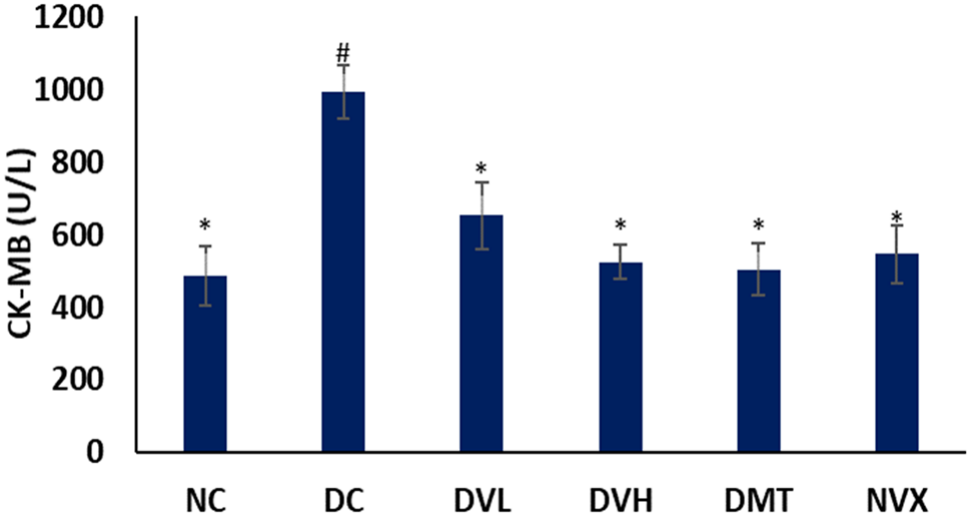
Serum CK-MB concentrations of experimental groups. Value = mean ± SD; n = 5. *Statistically significant (*p* < 0.05) to DC; #Statistically significant (*p* < 0.05) to NC. NC = normal rats, DC = diabetic control, DVL = diabetic rats + vanillin (150 mg/kg b.w), DVH = diabetic rats + vanillin (300 mg/kg bw), DMT = diabetic rats + metformin (200 mg/kg b.w), and NVX = normal rats + vanillin (300 mg/kg bw).

Induction of T2D significantly (*p* < 0.05) elevated serum levels of CK-MB as depicted in Fig. 11. Treatment with vanillin significantly (*p* < 0.05) reduced the level, with the high dose having a slight better activity. Both doses of vanillin compared favorably with DMT and NC groups.

**Figure 12:**
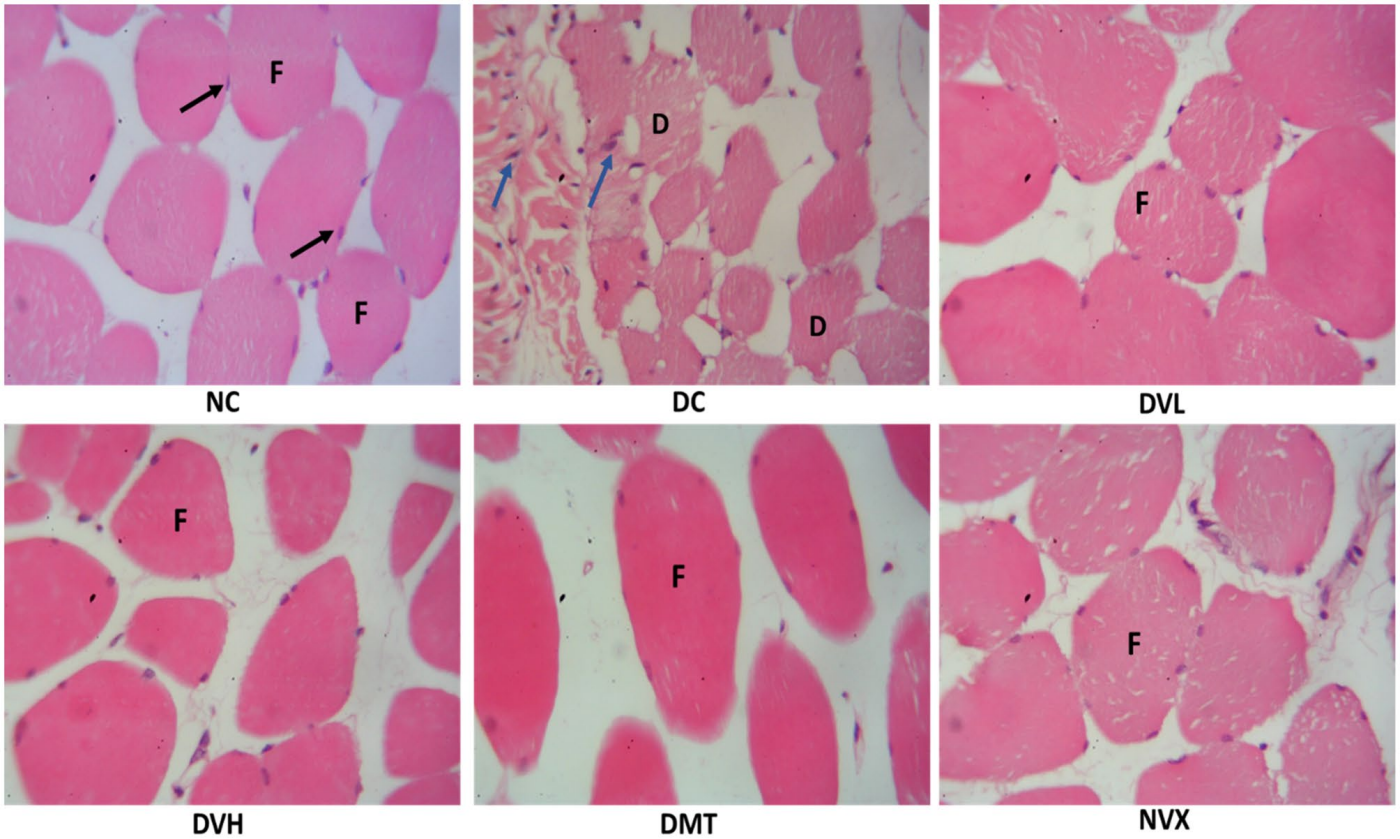
Histological changes in the muscles of experimental groups. magnification =  × 400. f—muscle fascicles; d—degenerating muscle fibres; black arrows—myocyte nuclei; blue arrows—inflammatory infiltrate. NC = normal rats, DC = diabetic control, DVL = diabetic rats + vanillin (150 mg/kg b.w), DVH = diabetic rats + vanillin (300 mg/kg bw), DMT = diabetic rats + metformin (200 mg/kg b.w), and NVX = normal rats + vanillin (300 mg/kg bw).

As shown in Fig. 12, histological analysis reveals that normal control (NC) group had normal muscle histology with homogenously distributed polygonal-shaped fascicles in transverse section and nuclei of myocytes lying at the periphery. However, this histology was modified on induction of T2D as indicated by several degenerating muscle fibres with obvious fibre disintegration and cytoplasmic vacuolations as well as inflammatory infiltrates. Treatment with the two doses of vanillin led to the mitigation of the T2D-induced histological insults as depicted by the improved muscle histology with mostly intact muscle fibres.

**Figure 13:**
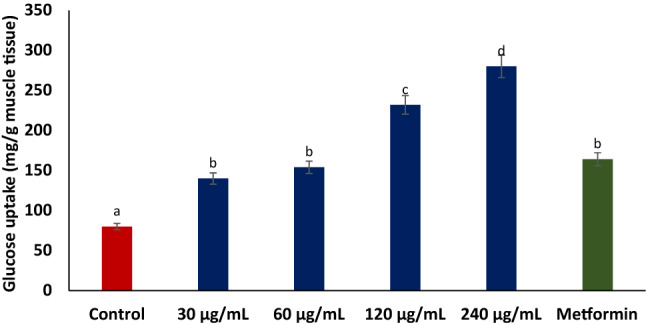
Effect of vanillin on glucose uptake in isolated rat psoas muscle. Data = mean ± SD; n = 3. ^a-d^Values with different letters above the bars for a given concentration are significantly (*p* < 0.05) different from each other.

Incubation of vanillin with isolated psoas muscle in the presence of glucose significantly (*p* < 0.05) enhanced glucose uptake as shown in Fig. 13. The activity was dose-dependent and compared favorably with metformin.

**Figure 14:**
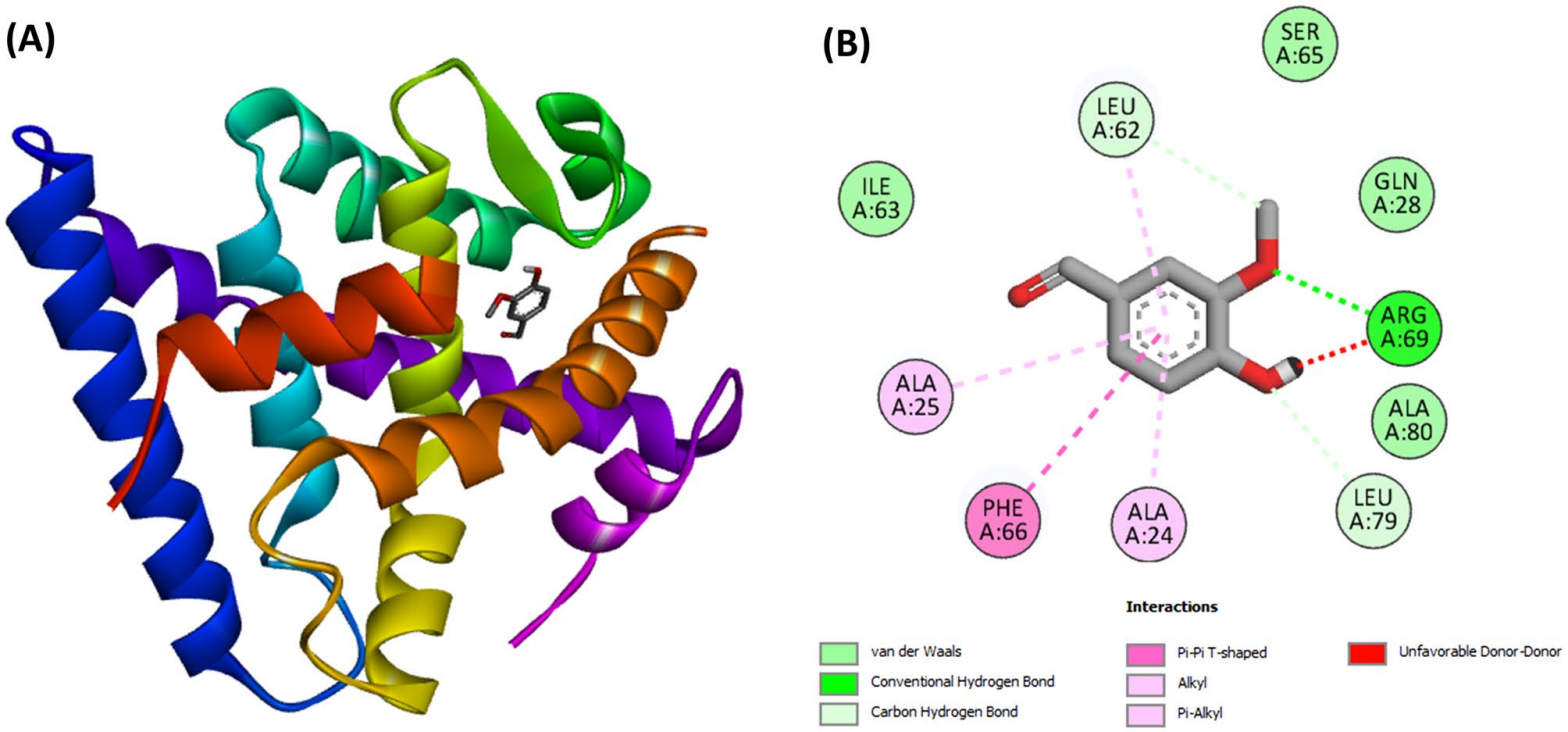
(**A**) 3D and (**B**) 2D representations of docked complexes of the active site of GLUT4 with vanillin.

Docking studies revealed a potent molecular interaction of vanillin with GLUT4 as shown in Fig. 14A, B, with a binding energy of − 6.2 kcal mol^−1^.

## Discussion

The skeletal muscle performs essential role in glucose homeostasis, and greatly affected by insulin resistance in T2D leading to muscle dysfunction and myopathy^11^. Most antidiabetic therapy is targeted at improving muscle function, glucose uptake and utilization^5,6^. Although the antidiabetic properties of vanillin have been reported, there is still a dearth on its effect on muscle metabolism in T2D. This study reports the ability of vanillin to improve muscle glucose-lipid metabolic switch while mitigating activities implicated in muscle dysfunction in T2D.

Oxidative stress is a key pathomechanism in T2D and its complications as it mediates insulin resistance^55,56^. Elevated oxidative stress incidence has been reported in muscles of diabetics, and has been linked to muscle wastage, dysfunction and impaired glucose uptake^6,11^. Oxidative stress is depicted in this study by the reduced levels of GSH, SOD and catalase activities in the muscle tissues and may be attributed to hyperglycemia-induced generation of free radicals. Increased generation of free radicals particularly superoxide (O_2_^•−^) in diabetic muscles has also been linked to glucose-activated NAD(P)H oxidase activity^57^. The increased MDA level (Fig. 2D) further depicts a lipid peroxidative effect on induction of T2D. The exacerbated levels of NO in muscles of untreated diabetic rats (Fig. 3) may insinuate increased generation of peroxynitrite (ONOO^-^) in the presence of excess O_2_^•−^ owing to suppressed SOD activity (Fig. 2B)^2,5^. Increased muscles levels of MDA and ONOO^−^ have been implicated in the impairment of insulin-mediated PI3-K, IRS-1 and Akt leading to downregulation of GLUT4^58^. Thus, suggesting impaired uptake and availability of glucose for muscle energy production. The elevated GSH level, SOD and catalase activities, and suppressed levels of MDA and NO following treatments with vanillin (Figs. 2 and 3) therefore indicate an antioxidative effect of the phenolic against muscle oxidative imbalance in T2D. This corroborates earlier reports on the potent antioxidant properties of vanillin^59,60^ and the protective effect of phenolics against muscle oxidative stress^5^.

The role of acetylcholine in skeletal muscles have been reported and has been linked to neuromuscular activities, muscle contraction and glucose uptake^61,62^. The increased acetylcholinesterase activity in muscles of diabetic rats (Fig. 4) suggests depleted levels of acetylcholine on induction of T2D. Thus, implying an occurrence of cholinergic dysfunction which corroborates previous reports on impaired cholinergic activities in diabetes^63^. The suppressed acetylcholinesterase activity in muscles of vanillin-treated groups (DVL and DVH) therefore indicates increased muscle acetylcholine, which further suggests improved neuromuscular functions and glucose uptake. Thus, implying an improved muscle cholinergic function. This corroborates previous reports on vanillin as a potent acetylcholinesterase inhibitor^13^.

Impaired purinergic enzymes activities have been implicated in muscular purinergic dysfunction leading to impaired contraction and glucose uptake^13,14^. The increased activity of ATPase, with concomitant suppressed ENTPDase and 5′NT activities in muscles of diabetic rats (Fig. 5A–C) indicate an occurrence of purinergic dysfunction, depicting suppressed levels of muscles’ ATP and adenosine following the induction of T2D. These modifications also imply a disturbance in muscle energy metabolism as these nucleotide and nucleoside are major substrates for energy metabolism^64,65^. ATP has also been reported for its role in muscle glucose uptake via translocation of GLUT4 and activation of P2 purinergic receptors^15,66^. The ability or vanillin to mitigate purinergic dysfunction in diabetic skeletal muscles is depicted in the present study by the suppressed ATPase activity, and elevated activities of ENTPDase and 5′NT in muscles of the phenolic-treated rats (DVL and DVH). These reversed activities further insinuate improved muscle glucose uptake and energy homeostasis. This corroborates earlier reports on the ability of vanillin and other phenolics to modulate the activities of purinergic enzymes^5,23^.

Impaired glucose metabolism in skeletal muscle leading to hyperglycemia has been implicated as a major patho-mechanism of T2D^67^. In normal muscles, glucose transported into the muscle undergoes glycolysis and subsequently to generation of ATPs^68^. This glycolytic flux plays a vital role in the regulation of muscle contractile function, with phosphofructokinase being an important regulatory enzyme^68,69^. This glycolytic flux is however impaired in muscles of T2D as depicted in the present study by the elevated FBPase (Fig. 6B)^70^, a major enzyme involved in gluconeogenesis. The elevated activities of G6Pase and glycogen phosphorylase (Fig. 6A, C) further indicates a glycogenolytic effect as these enzymes participate in the hydrolysis of glycogen to glucose. This is further depicted by the elevated muscle amylase activity in the untreated diabetic rats (Fig. 6D) as the enzyme also catalyzes the breakdown of muscle glycogen, thus leading to elevated muscle glucose content. The depleted muscle glycogen content in the DC group (untreated diabetic rats) (Fig. 7) may therefore be attributed to the elevated activities of G6Pase, glycogen phosphorylase and amylase. The increased gluconeogenic and glycogenolytic fluxes indicates a decreased generation of ATPs and further depicts a perturbed energy homeostasis. This correlates with the altered purinergic enzyme activities (Fig. 5A–C). The elevated activities of these glucose metabolizing enzymes may insinuate glucotoxicity which can trigger the generation of free radicals via oxidation of glucose to O_2·_^−^ and ketoaldehydes^71^. The excess glucose can also act as substrates for protein kinase C, hexosamine, AGE and polyol pathways^72^. Therefore, the decreased activities of fructose-1,6-biphosphatase, glucose-6-phosphatase, glycogen phosphorylase and amylase activities in the vanillin-treated groups (DVL and DVH) indicate restoration of glycolysis and glycogenesis, which insinuates an improved energy homeostasis arising from increased generation of ATP. This is further depicted by the elevated muscle content of glycogen.

Dysregulated lipid metabolism in muscles has been implicated as a key pathomechanism of muscle dysfunction in T2D^73^. This is depicted in this study by the elevated lipase activity (Fig. 8) and concomitant altered FFA metabolites (Table 1) and their distribution (Fig. 9) in muscles of the untreated diabetic rats (DC). The elevated lipase activity suggests hydrolysis of muscle triglyceride to FFAs, which is utilized as alternative substrates for energy production^5^. Furthermore, exacerbated FFAs level induces gluconeogenesis and glycogenolysis as a compensatory mechanism for depleted glucose level. Thus, suggesting a lipid metabolic switch over glucose in the diabetic muscles which corroborates Randal’s hypothesis of suppressed glucose utilization in states of exacerbated FFAs accumulation^74,75^. The deactivation of fatty acid biosynthesis, glycerolipid metabolism, fatty acid elongation in mitochondria, and fatty acid metabolism pathways (Fig. 10 and Table 2) further depicts dysregulated muscle lipid metabolism in T2D. Alterations in these pathways particularly, fatty acid elongation in mitochondria has been linked to suppressed glucose utilization and redox imbalance^76,77^. Impaired mitochondria fatty acid synthesis has also been implicated in loss of the electron transport chain and blockage of skeletal myoblasts differentiation *in vitro*^77^. Thus, suggesting that the deactivation of these pathways contributes to altered energy homeostasis and oxidative stress in diabetic muscles leading to their dysfunction. The reduced lipase activity (Fig. 8) restored FFAs metabolites (Table 1) and reactivated pathways (Table 2 and Fig. 11) in T2D rats treated with low dose of vanillin (DVL) indicates the ability of the phenolic acid to improve muscle lipid metabolism at low dose. Thus, suggesting a metabolic switch to glucose for muscle energy production which correlates with the suppressed gluconeogenesis and glycogenolysis as depicted by the decreased activities of G6Pase, FBPase and glycogen phosphorylase (Fig. 6A–C). The altered metabolites and deactivated pathways in muscles of T2D rats treated with high dose of vanillin (DVH) suggests that the phenolic maybe toxic at higher doses. This is further portrayed in normal rats administered high dose of vanillin (NVX).

Inflammation has been implicated in the pathogenesis of muscle dysfunction and myopathy in T2D^11,15^. The elevated serum level of CK-MB in muscles of untreated T2D rats (DC) (Fig. 11) indicates an inflammatory effect on induction of T2D. Besides cardiac muscles, creatine kinase-MB are found in skeletal muscles and are released into the serum following an inflammation of muscle cells. The decreased serum level in vanillin-treated rats therefore indicates an anti-inflammatory effect of the phenolic against T2D-induced muscle inflammation. This is in line with previous reports on the anti-inflammatory properties of vanillin^60^.

Changes in the muscle histology of untreated T2D rats (Fig. 12) as depicted by disintegrated fibres, cytoplasmic vacuolations, and inflammatory infiltrates further indicates an inflammatory effect which may be responsible for the leakage of CK-MB to the blood (Fig. 11). These histological insults indicate an occurrence of atrophy, myopathy and sarcopenia which are major attributes of muscle dysfunction in T2D^11^. Oxidative stress and inflammation arising from hyperglycemia and insulin resistance have been implicated in the pathogenesis of these insults^11^. The restored muscle histology in the vanillin-treated groups (DVL and DVH) therefore indicates the therapeutic effect of the phenolic against muscle dysfunction and myopathy in T2D.

The ability of vanillin to facilitate muscle glucose uptake in isolated psoas muscle (Fig. 13) indicates its ability to improve muscle glucose utilization. Thus, corroborating its effect on T2D-exacerbated activities of glucose metabolizing enzymes (Fig. 6A,D) and muscle glycogen content (Fig. 7). Facilitation of muscle glucose uptake is a major strategy in treating and managing T2D and its complications as seen with the biguanides^17,18^. This therapeutic mechanism has been attributed to their ability to upregulate GLUT4 translocation^78^. The potent molecular interaction of vanillin with GLUT4 (Fig. 14A,B) may indicate its ability to upregulate the transporter, which may also depict its glucose-uptake stimulatory mechanism. However, this is a computational study and therefore further wet studies are required to elucidate the effect of vanillin on GLUT4 expression in diabetic muscles.

## Conclusion

These results suggest the therapeutic effect of vanillin against muscle dysmetabolism and dysfunctions in T2D as depicted by its ability to mitigate oxidative imbalance, inflammation, cholinergic and purinergic dysfunctions, while modulating glucose-lipid metabolic switch and maintaining muscle histology. Thus, suggesting vanillin as a potent ingredient in the development of adjunct therapies for the treatment and management of T2D. Further studies are however recommended on the effect of vanillin on signaling molecules involved dysregulated muscle metabolism in T2D.

## Supplementary Information

Supplementary Information.
