# Supplementary material for: Vanillin modulates activities linked to dysmetabolism in psoas muscle of diabetic rats
Source: Sci Rep. 2021 Sep 21;11:18724. doi: 10.1038/s41598-021-98158-7 (PMC8455626; doi:10.1038/s41598-021-98158-7)
Supplement: Supplementary file 1 — Supplementary Information. [file 41598_2021_98158_MOESM1_ESM.docx]

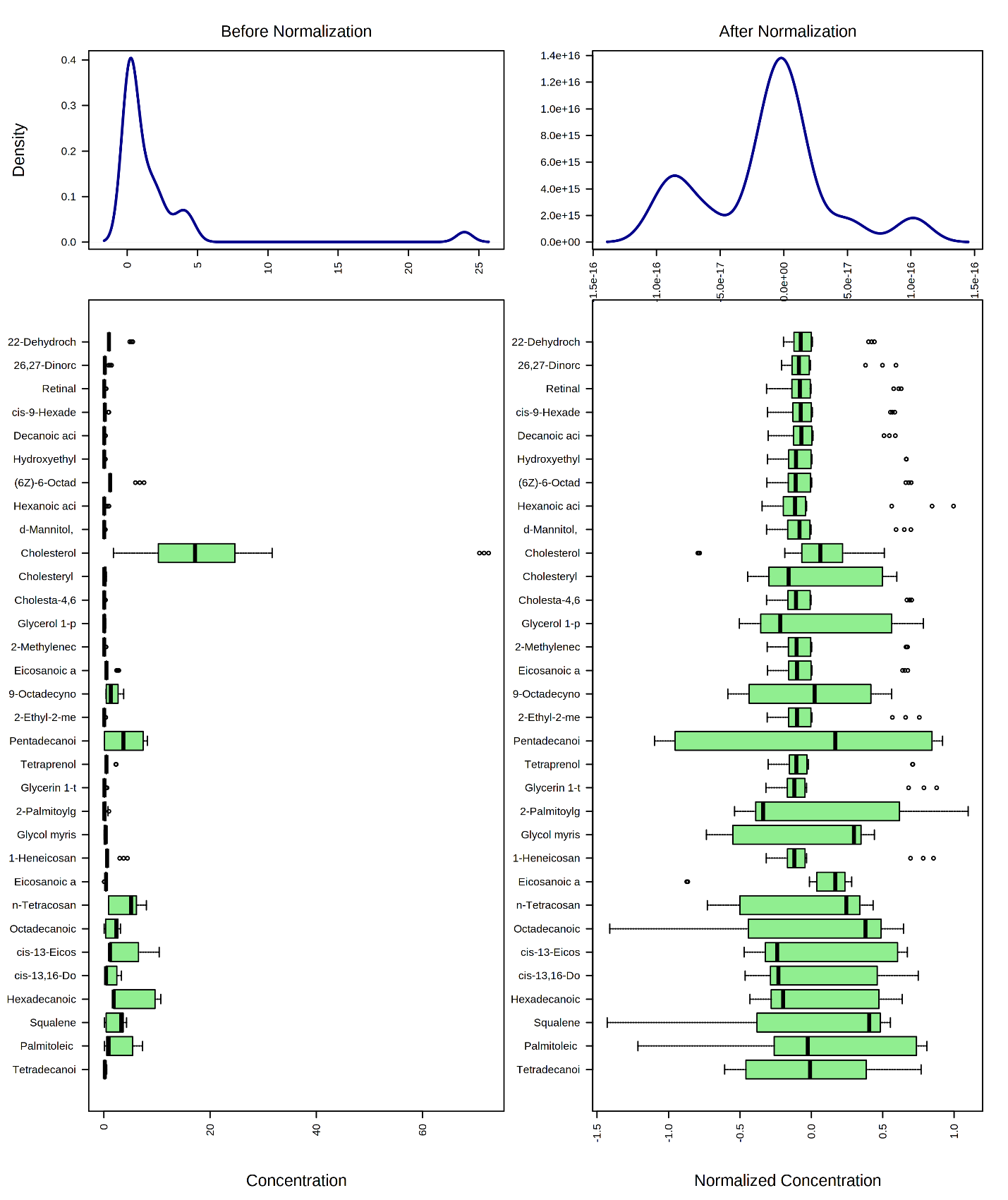


Fig. S1: Box plots and kernel density plots before and after normalization of GC-MS identified metabolites
